# Traditional Chinese Medicine Integrated with Chemotherapy for Stage II-IIIA Patients with Non-Small-Cell Lung Cancer after Radical Surgery: A Retrospective Clinical Analysis with Small Sample Size

**DOI:** 10.1155/2018/4369027

**Published:** 2018-07-25

**Authors:** Xueyu Zhao, Xiaojun Dai, Shanshan Wang, Ting Yang, Yan Yan, Guang Zhu, Jun Feng, Bo Pan, Masataka Sunagawa, Xiaochun Zhang, Yayun Qian, Yanqing Liu

**Affiliations:** ^1^Department of Combined Traditional Chinese and Western Medicine, Yangzhou University School of Medicine, Yangzhou, Jiangsu 225000, China; ^2^Yangzhou Cancer Research Institute, Yangzhou University, Yangzhou, Jiangsu 225009, China; ^3^The State Administration of Traditional Chinese Medicine Key Laboratory of Toxic Pathogens-Based Therapeutic Approaches of Gastric Cancer, Yangzhou University, Yangzhou, 225000, China; ^4^Medical and Pharmaceutical Institute, Yangzhou University, Yangzhou, Jiangsu 225000, China; ^5^Clinical Traditional Chinese Medical College, Yangzhou University, Yangzhou, Jiangsu 225000, China; ^6^Department of Physiology, School of Medicine, Showa University, Tokyo 142, Japan

## Abstract

**Objective:**

This study was designed to evaluate the clinical efficacy of combined traditional Chinese medicine (TCM) and conventional chemotherapy versus conventional chemotherapy in patients with stage II-IIIA non-small-cell lung cancer (NSCLC) after radical surgery.

**Methods:**

A retrospective cohort study was conducted in patients with stage II-IIIA NSCLC from Subei People's Hospital and Yangzhou Traditional Chinese Medicine Hospital in Yangzhou City of Jiangsu Province from 2012 to 2016. Patients were divided into two groups: the TCM user group (patients receiving treatment with integrated TCM and conventional chemotherapy) and the non-TCM user group (patients receiving conventional chemotherapy only). The two groups were compared for their median disease-free survival (DFS) and median overall survival (OS).

**Results:**

A total of 67 patients with stage II-IIIA NSCLC were enrolled between January 2012 and December 2016. The median DFS for the non-TCM user group was 601 days (95% confidence interval [CI], 375.7-826.3). The median DFS for TCM user group could not be calculated. However, log-rank analysis showed that the median survival time in the TCM user group was significantly longer than that of the non-TCM user group (P < 0.05). In addition, several significant risk factors were detected for predicting disease prognosis in patients with NSCLC, such as age, ECOG, lymphatic metastasis, and body mass index (BMI). For patients harboring these independent risk factors, the DFS of TCM user group was much longer than that of non-TCM user group (P < 0.05).

**Conclusion:**

Adjuvant therapy with TCM may reduce the rate of tumor recurrence and metastasis and prolong DFS of patients with stage II-IIIA NSCLC.

## 1. Introduction

As one of the most common malignant tumors, lung cancer is a leading cause of cancer-related death worldwide [1]. Non-small-cell lung cancer (NSCLC) accounts for 85% of all cases of lung cancer. Despite advancements in therapeutic approaches, the 5-year survival rate of lung cancer remains about 10%-15% [2]. At present, the comprehensive therapy regimen for NSCLC at early stage gives priority to surgery. Multiple factors lead to the low overall survival (OS) rates in NSCLC patients, such as late diagnosis, high tumor recurrence, and metastasis. Based on current methods of therapy, tumor recurrence and metastasis remain a great challenge for a full cure despite the excellent outcomes after standard treatments.

For patients with early stage NSCLC, surgery is the first therapeutic option. After operation, platinum-based chemotherapy or combined targeted therapy and chemotherapy are recommended as standard Western medicine treatments [3]. In addition, a comprehensive therapy regimen also includes radiotherapy and Traditional Chinese medicine (TCM). TCM is considered as an important complementary therapy with beneficial effects for patients with NSCLC by reducing toxic effects, improving the quality of life, and prolonging OS [4–6]. Lots of studies on antitumor mechanism of TCM have been reported. Xiong et al. [7] have found that a novel herbal formula can induce cell cycle arrest and apoptosis by suppressing the PI3K/AKT pathway in human lung cancer A549 cells. Pang et al. [8] found that the antitumor mechanism of Bu-Fei Decoction, a classical formula of traditional Chinese medicine (TCM), was related to interruption of the link between TAMs and NSCLC cells by inhibiting the expression of IL-10 and PD-L1 in vitro and in vivo. Shen SJ et al. [9] found that Yangfei Kongliu Formula, a compound Chinese herbal medicine, combined with cisplatin, can inhibit tumor growth synergistically, mainly through the TGF-*β*1 signaling pathway. Chinese herbal medicines (CHMs) have been used for thousands of years as adjuvant therapy and play an indispensable role in alternative medicine and cancer therapy [10]. TCM therapy mainly consists of Chinese herbal compound, which is composed of at least two or more traditional CHMs, containing herbal decoction, Chinese patent drug, and correlative injection.

TCM exhibits advantages in preventing tumorigenesis and attenuating toxicity, enhancing the therapeutic effect, and reducing risk of recurrence and metastasis [11]. When combined with adjuvant chemotherapy on postoperative early stage NSCLC patients, TCM led to partial relief of symptoms in addition to a reduction of side effects and adverse events caused by the chemotherapy regimens [12]. Although several systematic reviews and meta-analyses have evaluated the effectiveness of TCM treatment on lung cancer [4, 5, 13], their follow-up periods were relatively short with a lack of the comparison of disease-free survival (DFS) in NSCLC of stage II-IIIA. Therefore, we conducted a retrospective cohort study to evaluate the effect of TCM combined with conventional chemotherapy on DFS of patients with stage II-IIIA NSCLC.

## 2. Materials and Methods

### 2.1. Study Population

A total of 67 NSCLC patients were retrospectively enrolled, who underwent relevant treatments at Subei People's Hospital and Yangzhou Traditional Chinese Medicine Hospital between January 2012 and December 2016. The inclusion criteria for the study population were as follows: (i) patients were pathologically diagnosed with stage II-IIIA NSCLC after radical resection of pulmonary carcinoma; and the clinical TNM staging of II-IIIA NSCLC was based on 2009 Union for International Cancer Control (UICC,2009) staging of lung cancer; (ii) patients received integrated traditional Chinese and conventional chemotherapy or only conventional chemotherapy; (iii) the ages of patients were between 18 and 75 years old; and (iv) patients had good compliance. Clinical trials were excluded if they failed to meet the above criteria. In addition, the exclusion criteria were as follows: (i) NSCLC patients without clear pathological results; (ii) severe disease or dysfunction of heart, hepatic, kidney, or hematopoietic system; (iii) second primary malignancy; (iv) children, pregnant or lactating women, and psychiatric patients. All patients had complete clinicopathological data, including age, sex, smoking status, histological type, tumor size, and clinical stage. The enrolled patients were placed into either TCM user group (receiving treatment with integrated traditional Chinese and conventional chemotherapy) or non-TCM user group (receiving conventional chemotherapy only). The present study was approved by the Ethics Committees of Yangzhou University and both involved hospitals. In view of the retrospective nature of the study, a collection of informed consent was abandoned.

### 2.2. Medical Treatment

All patients in the non-TCM user group received only standardized chemotherapy according to the National Comprehensive Cancer Network (NCCN,2011) Clinical Practice Guidelines of NSCLC. All patients in the TCM user group received Chinese medicine therapy consisting of Chinese herbal decoction, Chinese patent drug, and correlative injection, together with standardized chemotherapy. Notably, the traditional Chinese medicine should be used for more than six months in the TCM user group. Standardized chemotherapy should be used for four cycles in two groups.

Based on the Chinese Medicine New Medicine Clinical Practice Guideline (Trial Implementation) (published by China Medical Science Press in 2002) and TCM theory of combination of disease and syndrome, combined with years of clinical observation and experience in our department, we summarized the main syndrome differentiation types at the postoperative stage of NSCLC as follows: pulmonary Qi deficiency, Qi and Yin deficiency, and stagnation of phlegm and blood stasis. They were determined by two senior physician. Pulmonary Qi deficiency manifests as the following: cough, shortness of breath, fatigue and weakness, spontaneous sweating, pale tongue, thin coating, and a weak pulse. Qi and Yin deficiency manifests as the following: cough, small amount of sputum, fatigue and weakness, dried mouth without polydipsia, spontaneous sweat, night sweat, reddish tongue or tongue with teeth imprints, and thready and weak pulse. Stagnation of phlegm and blood stasis manifests as the following: cough, constant phlegm, dark tongue, white and greasy coating, and an uneven pulse. Above all, the core pathogenesis (the pathological basis of tumor recurrence and metastasis) was the deficiency of vital Qi and internal toxin of cancer. On this basis, a therapeutic regimen was formulated based on the real diagnosis and treatment: the postoperative patients with pulmonary Qi deficiency syndrome adopted Shen Yi capsule (SDA approval number: Z20030044, Ginsenoside Rg3, orally administered of 20mg, two times each day (BID)); patients with Qi and Yin deficiency syndrome were treated with Yifei Qinghua granules (SDA approval number: Z20050851, whose ingredients included Radix Astragali seu Hedysari, Radix Codonopsis, Radix Glehniae, Radix Ophiopogonis, Semen Armeniacae Amarum, Bulbus Fritillariae Unibracteatae, and Herba Hedyotidis Diffusae; orally administered of 20g, two times each day (BID)); and Fufang Banmao capsule (SDA approval number: Z52020238, whose ingredients included Radix Astragali seu Hedysari, Panax ginseng, Aesculus wilsonii Rehd, and Chinese blister beetle; orally administered 0.75 g, two times each day (BID)), was used to treat patients with stagnation of phlegm and blood stasis syndrome. Additionally, all patients could choose to use oral Chinese medicine decoction added and subtracted from the standardized agent based on their core pathogenesis. The herbal treatment was suitable for the specific condition of each patient in accordance with syndrome differentiation [14]. There are three matched types of CHM decoction for the TCM user group in this study: patients with pulmonary Qi deficiency syndrome adopted LiuJunZi decoction, patients with Qi and Yin deficiency syndrome were treated with ShaShenMaiDong decoction, and patients with stagnation of phlegm and blood stasis syndrome were treated with SiWu decoction together with HuaTan decoction. The general outline of the TCM syndrome differentiation used and fundamental prescriptions given to the patients were described in Table 1. The major therapeutic principle was to strengthen the body resistance to eliminate pathogenic factors.

Decocting method is as follows: soak the herbs in water for 30 min with water level 1 cm above the herbs. First, boil with strong heat and then with gentle heat for about 20–40 minutes. Then, decant the decoction, repeat the above course, combine the decoction, and concentrate to 300 mL. Dosage and administration are as follows: one set of herbs per day, 150 mL each time, twice a day, and one hour after breakfast and supper.

### 2.3. Follow-Up

All patients were followed up carefully with hospital visits, telephone, or text messaging unless they were unable to be contacted. Physical examination, physical status, imaging examination (enhanced computed tomography for head and chest, bone scanning and ultrasonography for abdomen, etc.) and the time of tumor recurrence and metastasis, death, and OS were recorded. The ending time point of follow-up period was July 2017 or in the event that further data cannot be recorded.

### 2.4. Statistical Analysis

SPSS version 16 was used for statistical analyses (SPSS, Inc., Chicago, IL). In all tests, P < 0.05 was considered as statistical significance. Descriptive statistics were used to analyze the baseline characteristics of all patients. The rates of tumor recurrence and metastasis were assessed using the Kaplan-Meier method, with the log-rank test for evaluating significance. The Cox proportional hazard model was used to assess the prognostic values of variables.

## 3. Results

### 3.1. Patient Characteristics

All participants were enrolled from January 2012 to July 2016. There were two patients who were excluded due to the loss of follow-up, and the other 67 patients who met the inclusion criteria were eligible for analysis. Of these patients, 32 were in the TCM group and 35 were in the non-TCM group. In accordance with 2009 Union for International Cancer Control (UICC,2009) TNM staging system, 42 (62.7%) patients were at stage IIA- IIB and 25 (37.3%) patients were at stage IIIA. The present study included 47 men and 20 women, with a mean age of 59.9 years old (ranging from 45 to 75 years old). Forty-four (65.7%) patients, 21 (31.3%) patients, and one (1.5%) patient were diagnosed with adenocarcinoma, squamous-cell carcinoma, and adenosquamous carcinoma, respectively. There was no statistical significance between the groups regarding demographic and baseline characteristics (Table 2).

### 3.2. Survival of Patients

**Figure 1:**
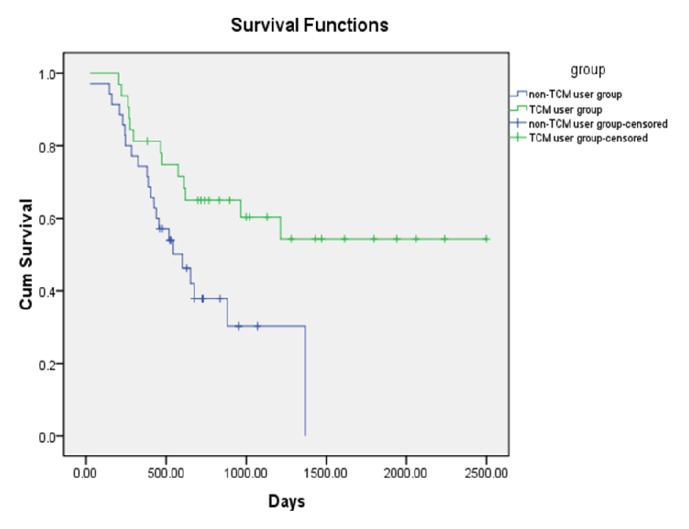
Comparison of DFS of patients according to different treatment.

Of all patients in the two groups, 35 patients suffered from tumor recurrence and metastasis. Ten patients passed away before July 2017. The median time of recurrence and metastasis for the non-TCM user group was 601 days (95% confidence interval [CI], 375.7-826.3). In comparison, the rate of recurrence and metastasis of TCM user group was 40.6%, and the median time of recurrence and metastasis could not be calculated. The log-rank analysis showed that the median survival time in the TCM user group was significantly longer than that of the non-TCM user group (P < 0.05) (Figure 1).

### 3.3. Prognostic Analysis

For multivariable analysis, the following parameters were considered as risk factors: age < 50 years old, the score of ECOG being 2, more than five lymphatic metastasis, BMI < 20, and occupation being cadre, with corresponding hazard ratios (HR) of 0.272 (95% CI = 0.141-0.525), 5.161 (95% CI = 1.932-13.791), 4.101 (95% CI = 1.531-10.989), 0.259 (95% CI = 0.91-0.734), and 4.426 (95% CI = 1.274-15.381), respectively. The above variables were independent prognostic risk factors (Table 3).

### 3.4. NSCLC with Stage II-IIIA Subgroups

**Figure 2:**
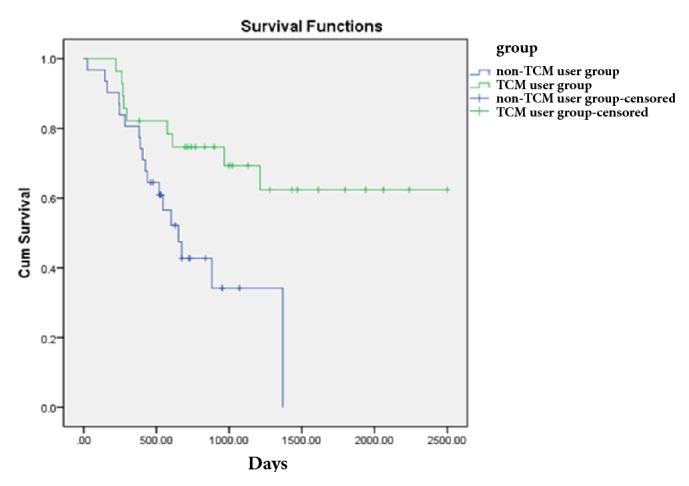
Comparison of DFS of patients with 0-1 prognostic risk factors according to treatment.

**Figure 3:**
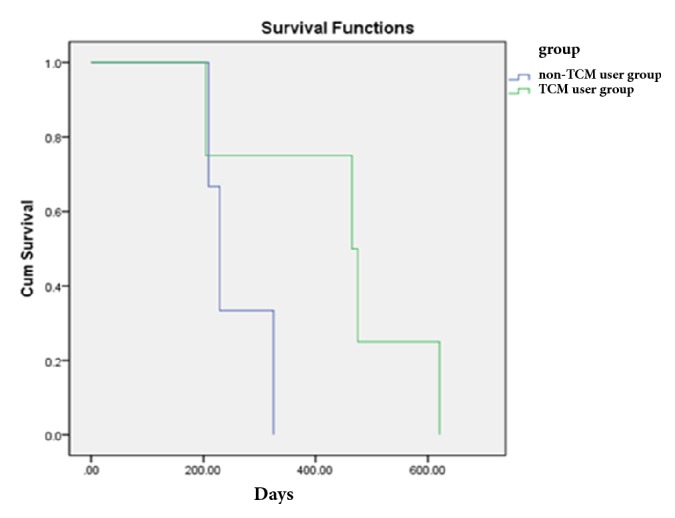
Comparison of DFS of patients with ≥2 prognostic risk factors according to treatment.

The early stage was further subdivided into several subgroups according to the presence of independent prognostic risk factors of age group, ECOG, lymphatic metastasis, BMI, and occupation as follows: no or one prognostic risk factor and two or more prognostic risk factors. Patients with no or one prognostic risk factor in the TCM user group harbored better prognostic outcome than the corresponding patients in the non-TCM user group. The median DFS time was 652 days (95% CI = 472.95-831.05) in the non-TCM user group. However, the rate of recurrence and metastasis of TCM user group was less than 50%, and the median DFS time could not be calculated. The log-rank test showed that the median survival time for the TCM user group was significantly longer than that in the non-TCM user group (P = 0.007) (Figure 2). When the prognostic risk factors reached two or more, the median DFS time was 465 days (95% CI = 199.42-730.58) in the TCM user group versus 229 days (95% CI = 196.99-261.00) in the non-TCM user group, which was not statistically significant between the two groups (P = 0.139) (Figure 3).

## 4. Discussion

NSCLC is a devastating disease with high incidence and mortality worldwide [15]. At present, surgical resection is the main treatment for early stage lung cancer. Platinum-based chemotherapy is considered to be necessary and effective after surgery for patients with early stage NSCLC, which might be able to reduce the risk of tumor recurrence [16]. In recent years, the concept of maintenance chemotherapy is questioned and revised because of the lack of survival benefit, increased toxicity, economic considerations, and poor quality of patients' life. About 50% of patients failed to complete the entire adjuvant treatment due to toxicity [17].

Therefore, whether Chinese medicine can reduce tumor recurrence and metastasis and prolong the survival of patients with NSCLC has become a hot topic in the field of Chinese medicine oncology. Recent studies have reported that combined with conventional chemotherapy, TCM showed superiority in relieving symptoms for patients with breast and gastric cancer, enhancing short-term efficacy, and improving quality of life [18, 19]. Moreover, no patients stopped TCM treatment attributed from the minimal adverse effects of herbs. In China, CHM is the most commonly used category of TCM [20]. Thus, it is worth evaluating the efficacy of TCM combined with conventional chemotherapy in the treatment of NSCLC, from a long-term perspective, to provide additional practice guidelines for treatment of lung cancer.

In the present study, the samples were selected in accordance with uniform inclusion criteria, exclusion criteria, and strict screening, which strengthened our evidence for research results. Moreover, TCM is recommended to be used for more than six months in clinical practice. Our findings showed that combined treatment of TCM and conventional chemotherapy could significantly prolong the DFS of patients with stage II-IIIA NSCLC in comparison with those treated with chemotherapy only. In addition, our research showed that the prognosis of TCM user group was better. Therefore, TCM, as adjunctive treatment, may be considered as an option of lung cancer treatment. There were five parameters for the prognostic values: age group, ECOG, lymphatic metastasis, BMI, and occupation. Lower age, higher ECOG score, more lymph node metastasis, and higher BMI led to higher rates of recurrence and metastasis. Also, compared to farmers, the risk of recurrence and metastasis for cadres was higher.

It should be noted that there are several limitations in this study. First of all, subgroup analysis of patients with stage II to IIIA according to clinical staging has not been done due to the limit of sample size. Secondly, the specimens of tumor focus and serum were not collected uniformly, and the molecular biological indexes were not studied and explored. Thirdly, the patients treated with a longer period of Chinese medicine (such as 1, 3, and 5 years) were not observed and followed up. Finally, in consideration of the feasibility of the project and the real diagnosis and treatment, the patients were not intervened by more individualized and comprehensive treatment with TCM.

In conclusion, since there are no published prospective, double-blinded trials of a TCM plus chemotherapy regimen following surgical resection of NSCLC, standardized large-scale, multicenter, and randomized double-blind controlled study should be used to carry out the clinical study of individualized treatment of patients with lung cancer in the future. Meanwhile, an evaluation system with Chinese characteristics should be established based on the clinical curative effects in view of effectiveness, safety, health economics, and ethics, improving authenticity and objectivity of research conclusions in order to present the superiority of Chinese medicine in cancer therapy as much as possible.

## Figures and Tables

**Table 1:** The most commonly used herbs per traditional Chinese medicine syndrome.

Chinese Name (Pinyin)	Pharmaceutical Name	English Name	Dosage (g)
Syndrome of Deficiency of pulmonary Qi(LiuJunZi decoction)			
Huangqi	Radix Astragali seu Hedysari	Membranous milkvetch root/ Mongolian milkvetch root	40
Dangshen	Radix Codonopsis	Pilose Asiabell Root /Moderate Asiabell Root/Szechwon Tangshen Root	15
Baizhu	Rhizoma Atractylodis Macrocephalae	Largehead atractylodes rhizome	10
Fuling	Poria	Indian buead	15
Chenpi	Pericarpium Citri Reticulatae	Tangerine Peel	6
Xingren	Semen Armeniacae Amarum	Bitter apricot seed	9
Syndrome of Deficiency of Qi and Yin(ShaShenMaiDong decoction)			
Huangqi	Radix Astragali seu Hedysari	Membranous milkvetch root/ Mongolian milkvetch root	40
Beishashen	Radix Glehniae	Coastal glehnia root	15
Maidong	Radix Ophiopogonis	Dwarf, lilyturf tuber, ophiopogon	15
Tiandong	Radix Asparagi	Cochinchinese asparagus root	15
Baihe	Bulbus Lilii	Lancelesf Lily Bulb / Greenish Lily Bulb	15
Beimu	Bulbus Fritillariae Unibracteatae	Unibract Fritillary Bulb	15
Syndrome of Stagnation of phlegm and blood stasis(SiWu decoction together with HuaTan decoction)			
Danggui	Radix Angelicae Sinensis	Chinese Angelica	15
Shudi	Radix Rehmanniae	Rehmannia Root	15
Chishao	Radix Paeoniae Rubra	Red Paeony Root	10
Chuanxiong	Rhizoma Chuanxiong	Szechuan Lovage Rhizome	10
Yiyiren	Semen Coicis	Coix Seed	20
Chenpi	Pericarpium Citri Reticulatae	Tangerine Peel	6
Banxia	Rhizoma Pinelliae	Pinellia Tuber	10
Ban Zhi Lian	Herba Scutellariae Barbatae	Barbed Skullcap Herb	15
Baihuasheshecao	Herba Hedyotidis Diffusae	Spreading Hedyotis Herb	15

**Table 2:** Patient base-line characteristics.

Characteristic	TCM user group(N=32)	non-TCM user group(N=35)	p
Sex, (%)			
Male	21 (65.62)	26(74.29)	
Female	11(34.38)	9(25.71)	0.439
Age, yr, (%)			
≤50	5(15.63)	5(14.29)	
50-65	20(62.50)	20(57.14)	
>65	7(21.87)	10(28.57)	0.820
Smoking status, (%)			
Never smoked	12(37.50)	12(34.29)	
<400	14(43.75)	12(34.29)	
≥400	6(18.75)	11(31.42)	0.474
ECOG performance status score—no. (%)			
0	0	2(5.71)	
1	27(84.38)	27(77.14)	
2	5(15.62)	6(17.14)	0.375
Tumor size, (%)			
<5cm	14(43.75)	17(48.57)	
≥5cm	18(56.25)	18(51.43)	0.693
Histologic diagnosis, (%)			
Adenocarcinoma	23(71.88)	21(60.0)	
Squamous-cell carcinoma	9(28.12)	12(34.28)	
Adenosquamous carcinoma	0	1(2.86)	
Other	0	1(2.86)	0.496
Clinical stage, (%)			
IIA	6(18.75)	13(37.14)	
IIB	14(43.75)	9(25.71)	
IIIA	12(37.50)	13(37.14)	0.167
modus operandi, (%)			
thoracotomy	17(53.13)	18(51.43)	
Thoracoscopy minimally invasive	14(43.75)	15(42.86)	
Combined	1(3.12)	2(5.71)	0.877

**Table 3:** Predictors of DFS in NSCLC patients.

**Variable**	**Univariate analysis**			**Multivariate analysis**		
	**HR**	**95**%**CI**	**P**	**HR**	**95**%**CI**	**P**
Age group (≤50 /50-65/≥65 years old)	0.505	0.292-0.871	0.014	0.272	0.141-0.525	0.000
ECOG (=0/1/2)	1.936	0.877-4.276	0.102	5.161	1.932-13.791	0.001
lymphatic metastasis(≥5/<5)	0.043	0.-4536.482	0.595	4.101	1.531-10.989	0.005
BMI(<20/20-24/>24)	0.351	0.145-0.851	0.021	0.259	0.91-0.734	0.011
Occupation (peasant/ worker/ cadre/ intellectual)	1.483	0.549-4.005	0.437	4.426	1.274-15.381	0.019
KPS (<80/≥80)	0.471	0.235-0.943	0.034			
Smoke (no/yes)	1.260	0.811-1.957	0.303			
Tumor size (≥5/<5)	1.115	0.570-2.180	0.750			
Clinical stages (IIA /IIB/IIIA)	1.139	0.748-1.735	0.543			
Treatment (TCM user/non-TCM user)	0.409	0.200-0.837	0.014			

## Data Availability

All data come from the medical records of the hospital and subsequent statistical analysis. These data will not be released to protect patient privacy.
